# Artificial Intelligence and Dentomaxillofacial Radiology Education: Innovations and Perspectives

**DOI:** 10.3390/dj13060245

**Published:** 2025-05-29

**Authors:** Daniel Negrete, Sérgio Lúcio Pereira de Castro Lopes, Matheus Dantas de Araújo Barretto, Nicole Berton de Moura, Ana Carla Raphaelli Nahás, Andre Luiz Ferreira Costa

**Affiliations:** 1 Postgraduate Program in Dentistry, Cruzeiro do Sul University (UNICSUL), São Paulo 01506-000, SP, Brazil; daninegrete75@gmail.com; 2 Department of Diagnosis and Surgery, São José dos Campos School of Dentistry, São Paulo State University (UNESP), São José dos Campos 12245-000, SP, Brazil; sergioluciolopes@gmail.com (S.L.P.d.C.L.); nicole.berton@unesp.br (N.B.d.M.); 3 Department of Oral and Maxillofacial Surgery, School of Dentistry, University of Sao Paulo, Sao Paulo 05508-000, SP, Brazil; matheusdabarretto@usp.br; 4 Department of Orthodontics, School of Dentistry, Guarulhos University (UNG), Guarulhos 07023-070, SP, Brazil; carlanahas@yahoo.com.br

**Keywords:** adaptive learning, artificial intelligence, clinical decision support, dental education, dentomaxillofacial radiology

## Abstract

Artificial intelligence (AI) is transforming dentomaxillofacial radiology education by enabling adaptive, personalized, and data-driven learning experiences. This review critically examines the pedagogical potential of AI within dental curricula, focusing on its ability to enhance student engagement, improve diagnostic competencies, and streamline clinical decision-making processes. Key innovations include real-time feedback systems, AI-guided simulations, automated assessments, and clinical decision support tools. Through these resources, AI transforms static learning into dynamic, interactive, and competency-based education. Additionally, this review discusses the integration of AI into formative assessment frameworks, such as OSCEs and mini-CEX, and its impact on student confidence, performance tracking, and educational scalability. Although primarily narrative in structure, this review synthesizes the current literature on dentomaxillofacial radiology education, supported by selected insights from medical radiology, to provide a comprehensive and up-to-date perspective on the educational applications of AI. Challenges (including ethical implications and other practical considerations) are addressed, alongside future directions for research and curriculum development. Overall, AI has the potential to significantly enhance radiology education by fostering clinically competent, ethically grounded, and technologically literate dental professionals.

## 1. Introduction

Artificial intelligence (AI) products are remodeling the radiological market. Recent market analyses project that the global AI diagnostics market, which includes radiology applications, will grow from USD 1.2 billion in 2023 to USD 5.4 billion by 2030, with a compound annual growth rate (CAGR) of 24.60% [1]. This wave of technology does not just change image interpretation, but also redesigns our approach to education [2].

AI-powered systems have changed the analysis of various dental imaging modalities, demonstrating notable precision in the analysis of both panoramic radiographs and cone beam computed tomography (CBCT) scans. These advancements significantly enhance learning outcomes and clinical results. Recent studies have shown that AI models achieve high accuracy rates in detecting periodontal bone loss, with sensitivity rates of approximately 0.99 for panoramic radiographs [3] and accuracy rates between 86% and 97% for dental image evaluation [4]. Furthermore, AI applications used for CBCT analysis have demonstrated even higher diagnostic accuracy, with pooled sensitivity and specificity for periapical lesion detection [5].

AI’s integration into radiology education delivers more than technological advancement because this technology creates customized learning paths and instant feedback, transforming how we train future dentomaxillofacial radiologists. This change will boost educational experiences and clinical skills for upcoming practitioners [6,7].

The integration of AI into dentomaxillofacial radiology education in undergraduate dental programs goes beyond mere technological advancement, as it changes the teaching methodology by creating personalized learning trajectories and providing real-time feedback [7]. This change is fundamental in transforming the way we educate dental students in dentomaxillofacial radiology. The result is a more engaging and effective learning environment that better equips dental students with radiological expertise, which is fundamental for modern dental practice. This AI-driven approach not only improves the educational journey, but also helps provide a deeper understanding of complex radiological concepts, eventually leading to more competent and confident general dental practitioners with strong backgrounds in dentomaxillofacial radiology.

In this review, we explore the transformative role of AI in teaching and learning about dentomaxillofacial radiology. We provide an overview of the current applications, opportunities, and challenges of integrating AI frameworks into the dentomaxillofacial radiology curriculum and propose educational strategies to effectively incorporate AI-driven resources.

## 2. AI as a Pedagogical Tool

The integration of AI into dentomaxillofacial radiology education marks a shift toward a precision model of dental training, enabling personalized instruction tailored to individual learning styles and needs [7]. AI-assisted platforms adapt to student progress in real time, identifying difficulties and offering targeted feedback or supplemental resources [8,9]. This adaptive approach promotes deeper engagement with complex radiological content and supports meaningful learning outcomes [10].

AI also enhances experiential learning by simulating clinical scenarios in a con-trolled, low-risk environment. Students can interact with virtual cases to diagnose conditions like periodontal bone loss, periapical lesions, and caries by using panoramic and CBCT images [11,12,13]. These simulations, supported by AI models with over 90% diagnostic accuracy in some studies [14,15], help bridge theoretical learning and clinical competence [16].

Moreover, AI facilitates immediate, objective feedback, which accelerates skill acquisition and fosters continuous, self-directed learning [17]. By tracking student progress over time, AI tools provide data-driven insights and recommend personalized learning pathways [8,18].

As dental programs scale up, AI addresses the need for consistent and high-quality training despite the increased image complexity and cohort size. It automates routine tasks such as image analysis and assessment, allowing instructors to focus on mentorship and higher-order teaching [19,20,21,22].

However, the adoption of AI in education creates challenges. Issues of data privacy and algorithmic fairness, as well as the need for faculty and infrastructure support, must be addressed to ensure ethical and effective implementation [23,24,25]. Despite these barriers, the pedagogical benefits of AI in fostering adaptive, scalable, and interactive radiology education are substantial [26].

### 2.1. Enhanced Learning Experience

AI enhances the learning process in dentomaxillofacial radiology by transforming passive content delivery into interactive and adaptive educational experiences. AI-powered platforms are capable of curating personalized case libraries that align with students’ knowledge, interests, and learning goals [27,28].

By simulating real-world scenarios and exposing students to a diverse range of imaging findings (including rare or subtle radiological features), AI systems help bridge the gap between theory and clinical practice. For example, algorithms can automatically select and show cases of atypical presentations or pathologies that may not be frequently encountered during undergraduate clinical rotations [28,29]. This ensures that students engage with a broader spectrum of diagnostic possibilities, thus fostering a deeper and more comprehensive understanding of radiological interpretation.

Moreover, AI systems can model and display the cognitive pathways used in diagnostic decision-making, offering step-by-step explanations and highlighting key radiological markers. This mirrors the way experts in radiology reason through cases, making tacit diagnostic knowledge more explicit and accessible to learners. Recent implementations of AI in radiology education have shown that this type of feedback can significantly improve the pattern recognition skills and reduce diagnostic errors among novice learners [29].

The integration of AI into self-directed learning environments also supports asynchronous education, allowing students to practice interpretative skills at their own pace, with immediate feedback and targeted reinforcement [6]. This makes AI not only a powerful teaching aid, but also a scalable and flexible solution for dental schools aiming to modernize radiology training.

### 2.2. Personalized Education Paths

In dental education, particularly in dentomaxillofacial radiology, students face the challenge of interpreting complex anatomical structures and radiographic variations that often require repeated exposure and individualized support [30]. AI offers a promising opening for personalizing this learning process, allowing the creation of adaptive educational environments that respond to each student’s specific needs and progression.

By continuously analyzing learners’ performance (e.g., diagnostic accuracy, time spent on image analysis, and recurring errors), AI-powered platforms can dynamically modify the content and difficulty level of the cases, radiographically and tomographically. For example, a student struggling with the identification of periapical lesions on CBCT scans can be directed to target resources, such as annotated 3D models, interactive quizzes with feedback, or similar clinical cases with progressive levels of complexity.

This form of personalization goes beyond static content delivery, as it creates learning paths that evolve with the student, thus reinforcing foundational knowledge while gradually introducing more nuanced cases. In the context of radiology, where pattern recognition and visual memory are key [31], this adaptive repetition strengthens clinical reasoning and prepares students for the diverse diagnostic scenarios they may encounter in actual practice.

Furthermore, AI-based recommendation systems integrated into digital learning platforms can assist students in accessing the most relevant resources from large image repositories or scientific literature based on their current learning focus (i.e., whether they are studying panoramic radiographs, CBCT anatomy, or pathology) [7,32]. This promotes autonomy and efficiency in learning by aligning with the principles of self-directed education, which are increasingly emphasized in modern dental curricula [33].

Finally, personalized AI-driven education in dentomaxillofacial radiology aligns with the broader goals of competency-based learning. It ensures that each student progresses toward clinical readiness at their own pace, with targeted reinforcement of weaker areas and recognition of strengths, thus offering a transformative approach to radiology instruction in dental schools [34,35].

### 2.3. Interactive Learning Modules

Interactive learning modules are essential components of AI-enhanced radiology education, offering multiple pedagogical advantages. Among their key features are real-time feedback on diagnostic interpretations, immersive virtual patient simulations, automated performance tracking, and high-fidelity clinical scenarios designed for skill development [17,28]. These elements collectively promote active, student-centered learning in dentomaxillofacial radiology.

AI-integrated educational platforms embedded within these modules continuously analyze students’ performance and adapt the content according to students’ individual learning trajectories [17,28]. By identifying specific gaps in knowledge or interpretation skills, the systems provide personalized support through targeted resources, such as annotated cases, supplementary readings, and guided practice exercises. This level of responsiveness helps ensure that learners receive the reinforcement they need in real time.

Before engaging in clinical practice, dental students can gain hands-on experience through AI-guided simulations that replicate real-life diagnostic and treatment planning scenarios. These virtual environments allow for repeated practice in a low-risk setting, thus enabling students to refine their skills and build confidence progressively [28]. Also, feedback is immediate, which allows students to monitor their progress closely.

Moreover, this interactive approach aligns with contemporary trends in health education, which emphasize experiential learning, learner autonomy, and engagement through simulation-based instruction [36]. By offering opportunities for repeated exposure to complex cases and constructive feedback loops, AI-driven learning modules bridge the gap between theoretical knowledge and clinical readiness by fostering a deeper and more lasting understanding of radiological principles and procedures [26,35,37].

**Figure 1:**
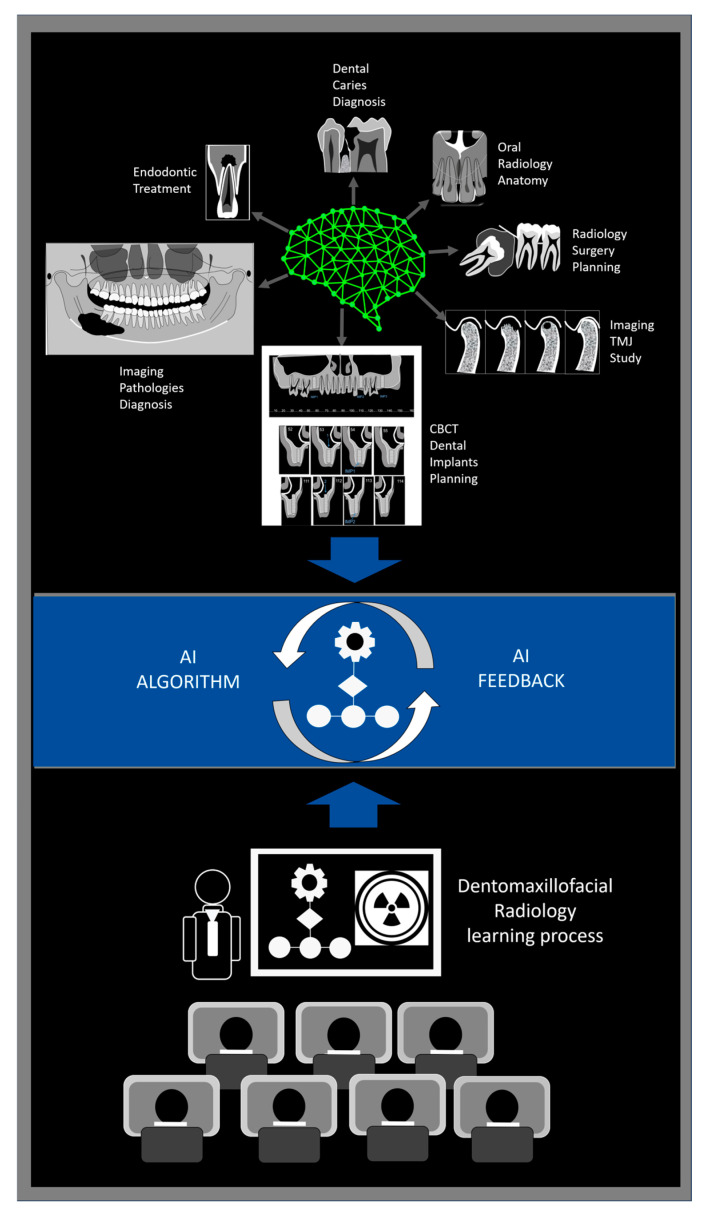
Schematic representation of an AI-driven feedback loop in dentomaxillofacial radiology education. AI algorithms assist students in performing diagnostic tasks such as caries detection, endodontic evaluation, pathology identification, implant and surgical planning, TMJ analysis, and anatomical interpretation. The system provides real-time and performance-based feedback, while educators facilitate critical appraisal and discussion of AI outputs. This dynamic, student-centered model promotes diagnostic accuracy through iterative learning, guided analysis, and continuous refinement of both student competence and algorithmic performance.

Figure 1 illustrates how AI-powered feedback loops support student learning in dentomaxillofacial radiology by integrating diagnostic tools, real-time feedback, and instructor guidance.

## 3. Transforming Clinical Decision-Making

AI–based clinical decision support (CDS) systems are increasingly transforming the landscape of dentomaxillofacial radiology by enhancing diagnostic precision and optimizing treatment planning. These tools integrate imaging findings with patient-specific clinical data to generate timely, evidence-based recommendations that assist dental professionals in making informed decisions [38].

In the radiological workflow, CDS systems can highlight suspicious anatomical features, suggest differential diagnoses, and recommend additional imaging when appropriate [39]. This is particularly relevant in the interpretation of complex CBCT datasets or subtle changes on panoramic radiographs, in which human variability may lead to diagnostic inconsistencies [39,40].

Moreover, by implementing clinical practice guidelines and structured decision pathways within their algorithms, CDS systems help standardize care and reduce cognitive load during routine and high-stakes decision-making [41]. In educational settings, such systems can also serve as real-time teaching tools, offering timely explanations and promoting the development of clinical reasoning skills among dental students [42].

The integration of AI-driven CDS into dentomaxillofacial radiology not only improves diagnostic accuracy and patient outcomes, but also contributes to safer, more efficient, and more consistent clinical workflows [38].

Although AI-powered clinical decision support systems offer valuable assistance in highlighting radiologic abnormalities, it is necessary to recognize that the field of dentomaxillofacial radiology has not yet reached the same level of diagnostic autonomy as broader medical radiology. At this stage, AI should primarily function as an alert system, drawing the clinician’s attention to potential findings that may require further evaluation. The final interpretation and diagnosis must remain the responsibility of the dentist, as diagnosis is inherently multifactorial, requiring the integration of clinical history, radiological findings, and, when appropriate, histopathological data. Failing to emphasize this could lead to overreliance on AI tools and compromise patient safety. As underscored by the Hippocratic principle “Primum non nocere” (“First, do no harm”), ethical and safe implementation of AI must always preserve the central role of the clinician in diagnostic reasoning and decision-making processes.

## 4. Automated Image Analysis

Deep learning algorithms have transformed the way dental images are analyzed, offering accuracy and efficiency to an extent which could not previously be achieved through manual interpretation alone. These models, particularly convolutional neural networks (CNNs), require substantial computational resources during their initial training phases, as they must process large annotated datasets to learn diagnostic features. However, once trained, they are capable of analyzing new images almost instantaneously, allowing for real-time integration into clinical workflows [43].

In the field of dentomaxillofacial radiology, AI-driven systems have demonstrated outstanding performance in the detection of various dental pathologies by using panoramic radiographs. Sensitivity rates as high as 0.99 have been reported for specific diagnostic tasks, including dental developmental stage classification and periodontal lesion detection [44]. Typically, the analytical pipeline begins with DICOM (Digital Imaging and Communications in Medicine) image conversion and preprocessing steps, such as contrast enhancement and noise reduction. The core detection stage often involves deep learning architectures (e.g., YOLOv5 or Faster R-CNN), which identify and localize disease markers with high precision [44].

By automating the identification of lesions, impacted teeth, periapical radiolucencies, or anatomical variations, these systems not only assist clinicians in early diagnosis, but also serve as valuable tools in dental education and clinical quality assurance [45,46].

Deep learning models have changed how we analyze images. Although these models require substantial computational power during training, they operate almost instantaneously with new datasets [43]. AI-powered systems show impressive results in detecting dental diseases from panoramic X-ray images, achieving sensitivity rates of about 0.99 [44]. Detection begins with DICOM conversion and advances to disease identification via trained faster regional convolutional neural networks [44].

## 5. Reporting Systems

The integration of AI into image reporting processes has significantly transformed how findings are documented in dentomaxillofacial imaging. AI-assisted reporting systems are capable of generating structured, concise, and standardized radiology reports, thus improving clarity and communication between radiologists and referring clinicians [27]. Recent studies have shown that AI-generated reports can reduce word and phrase counts by approximately 24%, while preserving diagnostic integrity and content [47].

These systems enhance report quality by automating the interpretation of diagnostic imaging data, which ensures consistent identification of key pathological findings. The use of standardized language and structured report templates reduces inter-rater variability, limits subjectivity, and enhances reproducibility. Furthermore, AI algorithms enable precise quantification of anatomical features and lesion dimensions, thus contributing to more objective reporting and facilitating longitudinal comparisons [48].

AI-enhanced reporting not only improves efficiency, but also promotes diagnostic consistency, reduces human error, and allows radiologists to dedicate more time to complex interpretative tasks and clinical decision-making [49].

## 6. Student Engagement and Adaptation

Structured support and systematic assessment are essential for allowing students to effectively adapt to AI-powered systems in dentomaxillofacial radiology education. Recent evidence shows that orientation seminars, guided training sessions, and formative assessments (e.g., mock exams) help students become acquainted with AI-integrated learning platforms, thus facilitating smoother transitions into digitally enhanced curricula [28].

### 6.1. Managing the Learning Curve

The learning curve associated with AI-assisted tools can be effectively addressed through structured training programs. Recent studies indicate that orientation sessions and structured AI education play a key role in preparing dental students for technology-enhanced assessments. Although formal training in AI remains limited in most dental curricula, students express a strong desire for greater integration of AI systems and feel more confident engaging with virtual classrooms and diagnostic platforms after guided instruction [50]. Initial engagement begins with specialized training modules that walk students through online interfaces, software navigation, and task workflows. This preparatory phase is followed by mock exams, which provide hands-on experience with AI-assisted evaluation environments, thus helping to reduce technical barriers that often hinder engagement in the early stages of digital transition [51].

### 6.2. Building Confidence

Student anxiety—particularly that related to unfamiliar digital interfaces and automated feedback systems—can negatively impact performance and engagement with AI applications. Studies have reported moderate anxiety levels among students when they are first introduced to AI-powered platforms, and these feelings are often tied to uncertainty about technical procedures or assessment accuracy [52]. Structured assessment strategies have proven to be effective in mitigating student anxiety associated with AI-assisted evaluations. For instance, integrating AI applications like ChatGPT into OSCEs has demonstrated potential in reducing trainee stress by providing realistic practice scenarios and real-time feedback. This approach not only enhances the consistency of assessments, but also alleviates the resource-intensive nature of traditional OSCEs, allowing educators to focus more on complex interpretative tasks and clinical decision-making [53].

Moreover, simulation-based training has been shown to enhance students’ confidence and competence in interpreting radiographic images and identifying complex anatomical structures, particularly in the context of oral and maxillofacial procedures. Interactive sessions contributed to improving the recognition of anatomical landmarks, supporting the value of targeted educational strategies in radiology instruction [54]. These findings emphasize the importance of progressive exposure and feedback to build students’ competence and self-assurance in AI-assisted radiology training.

### 6.3. Skill Assessment

The development of clinical competence in dentomaxillofacial radiology is strongly dependent on consistent performance tracking and provision of timely formative feedback [55]. Among the available evaluation tools, the mini-clinical evaluation exercise (mini-CEX) has gained importance as an effective workplace-based assessment instrument. When applied to AI-assisted environments, the mini-CEX facilitates real-time observation and evaluation of diagnostic reasoning by offering valuable insights into the students’ clinical decision-making processes. Students have rated this tool positively, citing its clarity, relevance to authentic clinical contexts, and its contribution to the development of structured interpretative skills [56].

Progressive improvements in assessment metrics have been observed throughout AI-integrated training programs. Students consistently reported increased efficiency and shorter evaluation lengths through successive seminars. This indicates not only growing familiarity with the technology, but also enhanced cognitive fluency in interpreting complex imaging cases. Structured, feedback-rich environments appear to accelerate the learning curve, particularly in tasks involving panoramic radiograph interpretation, in which learners showed measurable gains in both accuracy and reporting quality [57].

AI-assisted assessment environments have demonstrated measurable educational benefits, particularly when paired with structured feedback methods. In a recent randomized controlled trial [57], it was found that dental students who received structured, targeted feedback (comparable to what AI-supported platforms can deliver) achieved significantly higher diagnostic accuracy in panoramic radiograph interpretation than those who received only general comments. These findings suggest that formative, performance-specific guidance plays a key role in distinguishing between varying levels of clinical proficiency, thus reinforcing the educational value of AI-integrated evaluation systems.

Furthermore, reliability scores were close to the optimal thresholds when AI applications were incorporated into examinations, suggesting that these platforms can serve as viable and dependable alternatives to traditional in-person testing formats [53,58].

Beyond diagnostic accuracy, students highlighted improvements in soft metrics such as task clarity, appropriate difficulty calibration, and alignment with real-world clinical decision-making. Meta-evaluations of AI-integrated learning modules confirmed enhanced student engagement, improved execution time, and positive perceptions regarding assessment fairness and applicability to clinical contexts [16].

Taken together, these findings underscore the capacity of well-designed AI-supported assessment systems to not only track performance effectively, but also to foster learner autonomy and confidence. When paired with scaffolded instructional strategies and adaptive feedback mechanisms, these technologies can substantially elevate the quality and efficiency of radiological training in dental education.

## 7. Enhancing Patient-Centered Care

AI in dentomaxillofacial radiology has significantly advanced the delivery of patient-centered care. By increasing diagnostic precision and enabling tailored treatment strategies, AI contributes to more individualized and effective clinical decision-making. Advanced algorithms are capable of analyzing complex dental imaging data with high accuracy, including panoramic radiographs and CBCT, facilitating earlier detection of pathologies and more accurate anatomical assessment.

These improvements not only enhance clinical efficiency, but also support the development of personalized treatment plans based on the unique radiological and clinical characteristics of each patient [27]. AI-based systems can process large volumes of patient data and imaging findings to generate evidence-based recommendations, thus helping practitioners present clearer, more informed options to their patients.

Additionally, the incorporation of AI into radiological workflows strengthens communication between clinicians and patients. Automated reporting tools and explainable AI systems can simplify technical findings by translating them into a more accessible language and improving the patient’s understanding. This fosters greater transparency, encourages shared decision-making, and builds stronger trust in the care process.

Recent literature has emphasized that AI frameworks contribute to patient satisfaction by promoting more predictable outcomes and reducing uncertainty associated with complex diagnoses and treatments [59,60]. As a result, the shift toward AI-supported patient-centered imaging represents a critical evolution in the way how radiological care is delivered in contemporary dental practice.

## 8. Research and Innovation Opportunities

AI applications are enabling more sophisticated data processing, enhancing diagnostic accuracy, and supporting the development of advanced clinical protocols. Recent studies demonstrate that AI performs remarkably well in dental imaging tasks, leading to statistically significant improvements in diagnostic performance and predictive accuracy [61]. Deep learning algorithms can identify subtle image features, thus reducing inter-rater variability and recognizing complex patterns that may not be easily detected by the human eye.

### Clinical Research Integration

AI-powered systems can generate clinical summaries, operative notes and reports with high readability and accuracy, thus supporting the educational process [62]. Also, AI-driven image interpretation systems are being integrated into dental curricula to allow students to engage with automated diagnostic frameworks with high accuracy rates for various dental pathologies [63]. These systems are particularly effective in the detection of maxillofacial cysts and tumors, early identification of carious lesions and periapical pathologies, and precise classification of radiological abnormalities. The convergence of AI with clinical and imaging data provides a formidable framework for innovation by facilitating translational research and enhancing both diagnostic processes and educational frameworks in dentomaxillofacial radiology [60].

## 9. Impact on Professional Development

The integration of AI into dentomaxillofacial radiology education is significantly influencing the professional development of future dental practitioners [22]. As AI becomes more prevalent in diagnostic workflows, dental professionals are expected to acquire a diverse skillset that encompasses clinical expertise, technological proficiency, data analysis, and ethical judgment.

Beyond the enhancement of technical competencies, AI contributes to the development of a more reflective and analytical mindset in dental students. Adaptive learning platforms and AI-assisted simulations offer personalized feedback that enables learners to monitor their own progress and address specific weaknesses [8]. As a result, new generations of dentists are emerging with a more comprehensive data-informed approach to patient care and are better equipped to integrate clinical judgment with digital technologies in a responsible and ethical manner [64].

## 10. Discussion

The application of AI to dentomaxillofacial radiology education marks a significant advancement in the modernization of dental training as educational programs face increasing demands for efficiency, scalability, and quality, with AI offering solutions that align with these goals.

AI-assisted simulations and case-based learning systems allow students to develop diagnostic proficiency in a low-risk environment, thus promoting deeper understanding and clinical readiness. Real-time feedback mechanisms and adaptive content delivery also support self-regulated learning and help tailor instruction for individual learners’ needs [42].

Moreover, AI’s influence extends beyond technical skills, as it fosters critical reflection as well as engagement with complex radiological concepts. The integration of AI into formative assessments, such as OSCEs and mini-CEX, provides instructors with valuable data on the students’ progress while helping them gain confidence in diagnostic reasoning. As noted by Misra & Suresh [53], AI-enhanced assessments not only improve reliability, but also reduce student anxiety and logistical constraints. Similarly, personalized feedback loops and learning analytics contribute to competency-based education models by reinforcing the shift toward student-centered curricula [16].

However, the successful adoption of AI tools requires paying attention to implementation challenges. These include the need for infrastructure, faculty development, curricular alignment, and ethical oversight. Educators must ensure that AI systems are used transparently, respect patient data privacy, and complement rather than replace human judgment. As highlighted in the recent literature, including Uribe et al. [63] and Heo et al. [60], sustained interdisciplinary collaboration and evidence-based guidelines are essential for maximizing the educational value of AI technologies.

Beyond educational environments, the growing use of AI in clinical contexts raises important considerations regarding patient care and ethical responsibility. Dental students trained with AI-enhanced tools must develop not only technical proficiency, but also an awareness of issues such as data privacy, informed consent, and algorithmic bias. As Uribe et al. [63] point out, integrating generative AI into healthcare education necessitates a commitment to ethical guidelines that prioritize transparency, fairness, and accountability. Likewise, Heo et al. [60] emphasize the importance of maintaining clinician oversight in AI-assisted diagnostics in order to ensure that patient care decisions remain grounded in professional judgment and individualized needs. Therefore, fostering ethical literacy alongside technological competence is essential to prepare future practitioners for the complexities of AI-supported clinical practice.

## 11. Conclusions

The growing integration of AI into dentomaxillofacial radiology education reflects a critical shift in how dental professionals are prepared for practice in the digital age. As AI increasingly influences diagnostic workflows and clinical communication, educational systems must evolve to ensure that students acquire not only technical fluency, but also the ethical, analytical, and collaborative competencies required to navigate AI-enhanced environments. Establishing comprehensive and flexible educational frameworks will require collaboration among dental educators, informatics experts, radiology faculty, and academic institutions. Although no single model will suit all contexts, a combination of formal instruction, case-based learning, and self-directed resources can bridge the current gap between AI innovation and its effective pedagogical deployment. By investing in thoughtful AI education today, institutions can lay the foundation for future dentists who are confident, capable, and conscientious stewards of emerging technologies.

## Data Availability

Not applicable.
